# Conformal transistor arrays based on solution-processed organic crystals

**DOI:** 10.1038/s41598-017-15518-y

**Published:** 2017-11-13

**Authors:** Xiaoli Zhao, Bing Zhang, Qingxin Tang, Xueyan Ding, Shuya Wang, Yuying Zhou, Yanhong Tong, Yichun Liu

**Affiliations:** Key Laboratory of UV Light Emitting Materials and Technology under Ministry of Education, Northeast Normal University, Changchun, 130024 P. R. China

## Abstract

Conformal transistor array based on solution-processed organic crystals, which can provide sensory and scanning features for monitoring, biofeedback, and tracking of physiological function, presents one of the most promising technologies for future large-scale low-cost wearable and implantable electronics. However, it is still a huge challenge for the integration of solution-processed organic crystals into conformal FETs owing to a generally existing swelling phenomenon of the elastic materials and the lack of the corresponding device fabrication technology. Here, we present a promising route to fabricate a conformal field-effect transistor (FET) array based on solution-processed TIPS-pentacene single-crystal micro/nanowire array. By simply drop-casting the organic solution on an anti-solvent photolithography-compatible electrode with bottom-contact coplanar configuration, the transistor array can be formed and can conform onto uneven objects. Excellent electrical properties with device yield as high as 100%, field-effect mobility up to 0.79 cm^2^V^−1^s^−1^, low threshold voltage, and good device uniformity are demonstrated. The results open up the capability of solution-processed organic crystals for conformal electronics, suggesting their substantial promise for next-generation wearable and implantable electronics.

## Introduction

Next-generation electronics require that devices with lightweight, flexibility, and portability, can be worn directly on the human body, or integrated into the existing life items. Conformal devices with good flexibility and elasticity, that enable intimate and noninvasive adherence on uneven surfaces, have shown a big potential in next-generation electronics, such as electronic skin, human activities monitoring, personal healthcare, and human-machine interface^1–6^. Previous work based on geometric designs, such as wavy^7,8^ and serpentine structures^9^, transfers the stiff-island devices onto prestrained stretchable substrates to impart conformability to electronics and presents the potential for a variety of wearable and implantable applications. However, the rigid nature of these active devices has limited device density, mechanical robustness, and wide applicability^10^. So far, the whole device with sufficient conformability remains scarce^10–14^, because the fabrication of such a device strictly requires a new design of device geometry and a relevant adopt of materials possessing elasticity and flexibility to eliminate strain-introduced wrinkles through electrostatic adherence. Only recently, Bao’s group has made some breakthrough progress on fabricating single conformal organic transistor with outstanding stretchable ability and electrical performance by successively transferring the prefabricated gate dielectric, semiconductor thin film, and source/drain electrode onto elastic substrates^10,11^.

Solution-processed organic crystal array offers great potential for achieving low-cost and large-scale production for flexible and conformal electronics^15–18^, because they combine the advantages of both solution technology and organic crystals as semiconductors. The organic crystal array can be easily obtained by low-temperature solution method, and the nature of single crystals can eliminate disorders and traps, which is beneficial for the development of high-performance organic electronic devices^19–22^. However, almost all the reported solution-processed organic crystals are fabricated on rigid or flexible plastic substrates, limiting their mechanical robustness and extensive applicability^15–22^. To date it is still a huge challenge for the integration of the solution-processed organic crystals into the conformal field-effect transistors (FETs) owing to a generally existing swelling phenomenon of the elastic materials^23^ and the lack of the corresponding device fabrication technology.

Here, we have designed a novel photolithography-compatible anti-solvent coplanar electrode with bottom-gate bottom-contact configuration, for integration of solution-processed TIPS-pentacene single-crystal array into conformal FETs. The resulting FET array presents excellently electrical properties with device yield as high as 100%, good device uniformity, low threshold voltage, and field-effect mobility up to 0.79 cm^2^V^−1^s^−1^. The obtained mobility is in class of the highest value among the reported bottom-contact TIPS-pentacene FETs. Our devices show the outstanding conformability to curved objects, and exhibit sensitive changes for different bending strains, indicating the strong potential applications in wearable and implantable electronics.

## Results and Discussions

**Figure 1:**
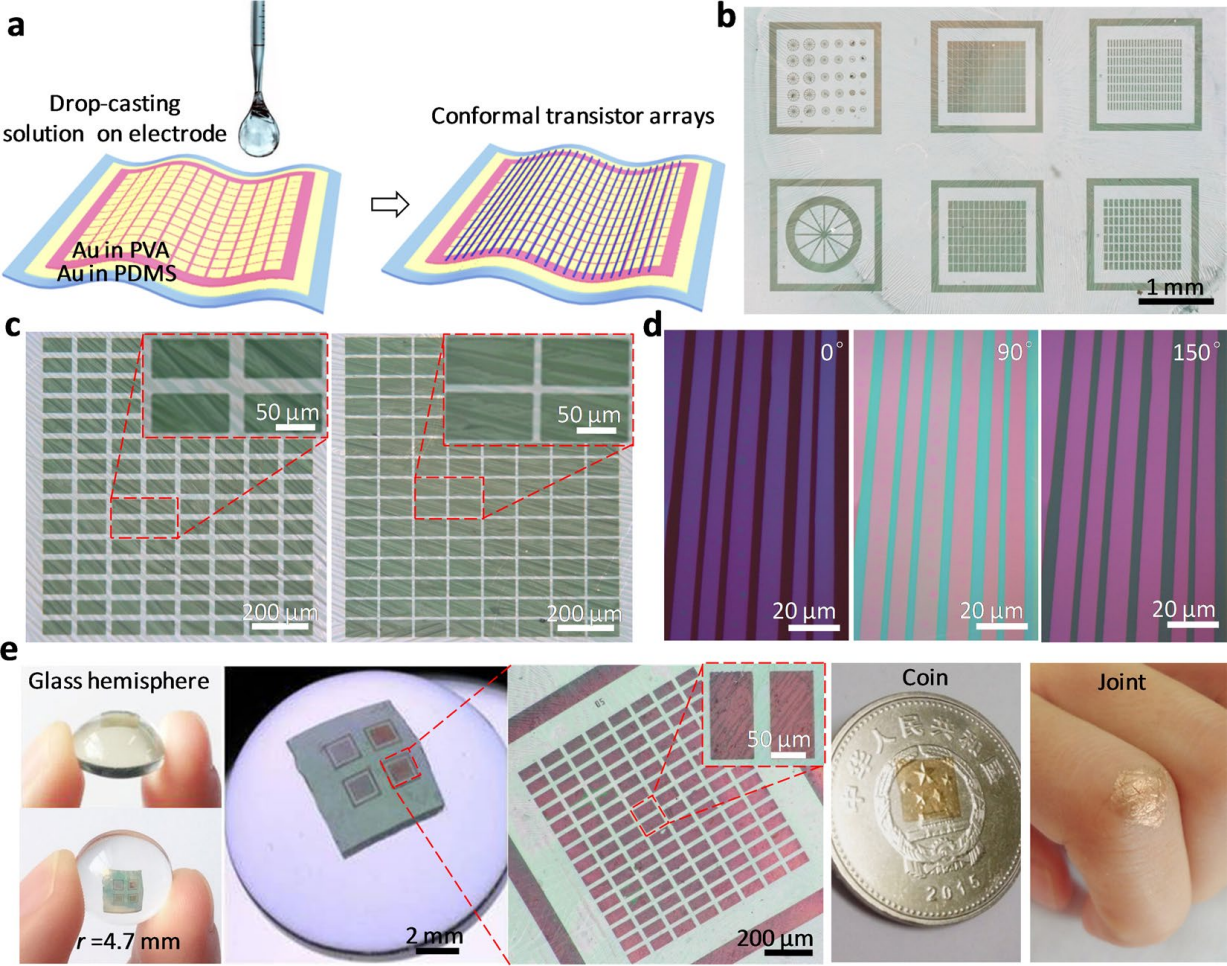
Conformal organic single-crystal FET arrays. (**a**) Fabrication schematic diagram of the conformal device arrays. The organic semiconductor solution is drop-casted on an anti-solvent conformal electrode with embedded source/drain/gate contacts. (**b**,**c**) Transmission optical microscopy images of a real large-scale TIPS-pentacene single-crystal FET pattern and two typical device arrays with magnified views. (**d**) Polarization optical microscopy images of TIPS-pentacene micro/nanowire array on PVA dielectric under angles of 0°, 90°, 150°, indicating the single-crystal nature of the oriented array. (**e**) Devices can well conform to different shaped objects, such as glass hemisphere, coin, and human joint. 3D optical microscopy images clearly show no bubbles or wrinkles on the whole surface of the curved device array adhered to 3D glass hemisphere.

Figure 1 illustrates the main idea and fabrication scheme of conformal organic single-crystal FET array. To integrate solution-processed organic crystal array into the conformal electronic devices, we develop a novel photolithography-compatible anti-solvent electrode fabrication technique (see Experimental Section). So far, it is challenging to integrate the solution deposition of organic semiconductors with the conformal electronics. The dilemma is that conformal electronic devices generally used the elastomer to achieve conformability, while most of organic solvents such as chloroform and chlorobenzene can cause the cured elastomer to swell into a quite large volume, and the diffusion of organic solution into the elastomer^23^. In our experiments, it was found that the swelling of PDMS occurred when organic solution was drop-casted onto PDMS surface, resulting in the formation of only few crystal array (see Figure S1, Supporting Information). In order to effectively avoid swelling phenomenon, an anti-solvent insulated material, i.e. polyvinyl alcohol (PVA), was selected as the dielectric layer, which possesses good flexible and film forming property, showing excellent conformability onto different shaped objects, such as glass hemisphere and human finger as shown in Figure S2. It is well known that PVA is soluble in water and insoluble in almost all the organic solvents such as gasoline, kerosene, vegetable oil, benzene, toluene, dichloroethane, carbon tetrachloride, acetone, ethyl acetate, methanol, ethylene glycol etc. Therefore, the cured PVA film do not produce any dissolved or swollen phenomenon when it was dipped into chloroform solution (Movie S1, Supporting Information). As schematically shown in Fig. 1(a), when a droplet of TIPS-pentacene/chloroform solution was casted onto the prefabricated anti-solvent electrode pattern, the evaporation of the solvent resulted in the formation of the large-scale TIPS-pentacene single-crystal FET array. It is worth mentioning that the electrode was fabricated by photolithography. Therefore, as shown in the optical microscopy image of Fig. 1(b), the large-scale transistor array with versatile electrode patterns can be easily obtained. Figure 1(c) clearly presents the two typical device arrays with different electrode patterns on one wafer. The grown micro/nanowire array with the length ranging from hundreds of micrometers to several millimeters can be oriented distributed on the electrode patterns. They can uniformly cross electrode patterns without fracture appearance. In order to gain insight into the nature of the grown TIPS-pentacene micro/nanowire array, the color of the micro/nanowire array is observed under the polarizing optical microscope. As shown in Fig. 1(d), the color along each micro/nanowire is uniform under a fixed polarization angle. When the different polarization angles are applied, the colors of micro/nanowire array change uniformly. These results indicate the single-crystal structure of an individual micro/nanowire and the anisotropic crystalline nature of the oriented micro/nanowire arrays caused by the molecular packing^24–26^.

As shown in Fig. 1(a), the Au gate electrode coupled with the elastic PDMS supporting layer ensures the conformability of the transistor array onto the curved surfaces. To examine the ability of the fabricated solution-based organic single-crystal FET array to conform onto uneven objects, we adhered the transistor array on different objects which can be rigid or flexible, smooth or rough, organic or inorganic. One edge of the device array initiates contact with the object surface, and then van der Waals force causes the device array to gradually wet along the curved surface without the external mechanical pressure to establish the contact. The stamp process is reversible and nondestructive, and the device array can be easily put on and taken off, even multiply transferred onto various objects. As shown in Fig. 1(e), our device array well conform to different objects, such as small glass hemisphere, uneven coin, and human joint, which illustrates the promising potential for communication, biology, healthcare, military, *etc*. The 3D optical microscopy images clearly show no bubbles or wrinkles on the whole surface of the curved device array on the glass hemisphere with radius of 4.7 mm. It confirms that the soft device array provides a conformal contact and adequate adhesion on the curved surface based on van der Waals interaction, which well meets the requirements of next-generation electronic devices.

**Figure 2:**
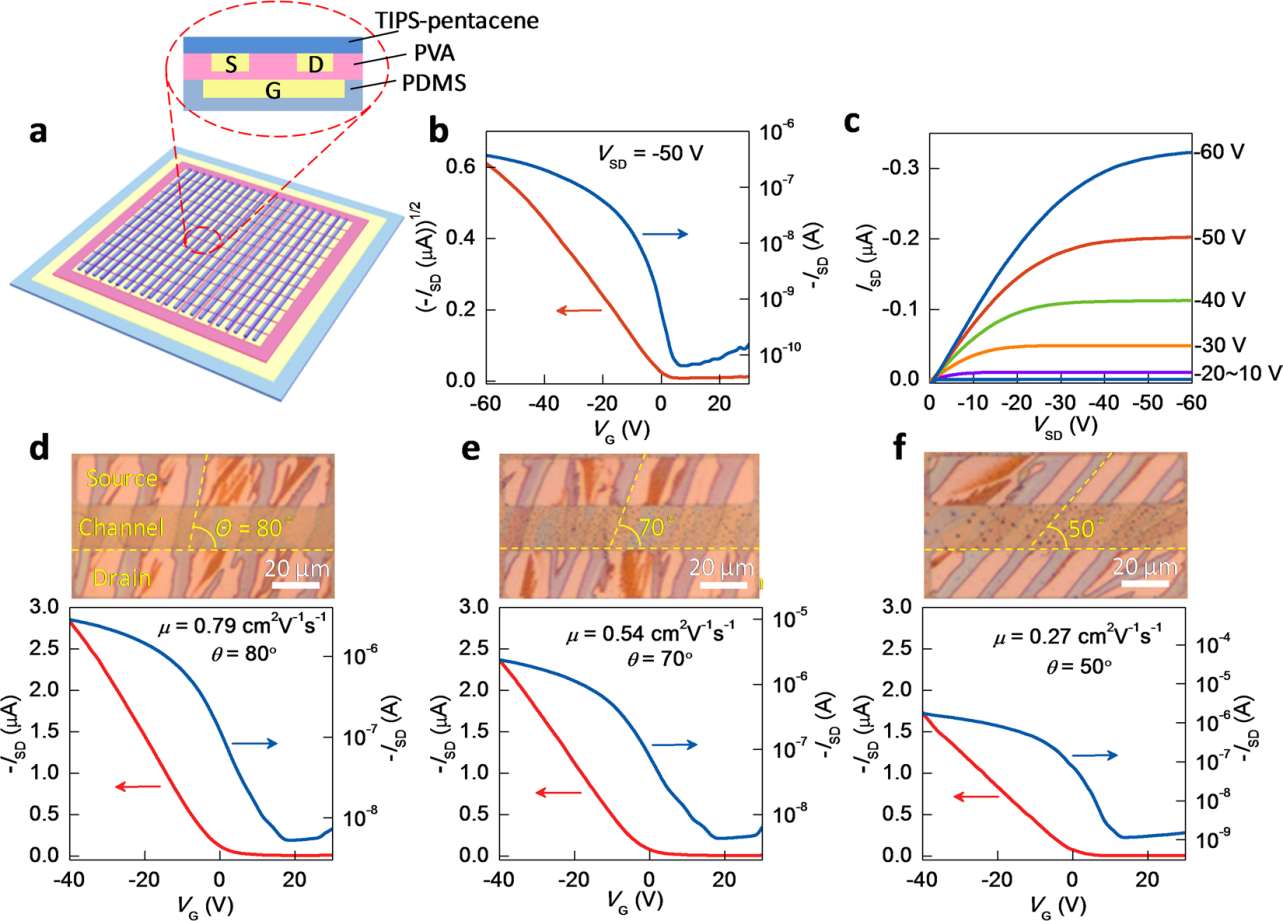
Effect of crystalline texture orientation on the field-effect performance of conformal organic single-crystal FETs. (**a**) Schematic and the corresponding cross-sectional diagrams of the device. (**b**,**c**) Typical transfer and output characteristics of the device measured in air at room temperature. (**d**–**f**) Optical microscopy images and the corresponding transfer curves of three devices with different oriented TIPS-pentacene micro/nanowire array with respect to the channel. A clear correlation between the field-effect mobility and the texture orientation is observed.

Figure 2 shows the electrical performance of the conformal TIPS-pentacene single-crystal micro/nanowire FETs. TIPS-pentacene was selected as the channel material in the conformal OFET array, owing to the fact that it is one of the most promising organic materials in desirable electrical performance and good solubility in common solvents^19,21,22^. Figure 2(a) clearly shows the schematic diagram and the corresponding cross-sectional image of the device. The typical transfer and output characteristics of the device are shown in Fig. 2(b) and (c). All transistors show the clear p-type characteristics. As we all know, in the growth process of organic micro/nanowires, the orientation of micro/nanowires is consistent with the direction of solution evaporation. Therefore, the conformal device array with different orientations can be easily obtained by controlling the position of dropping solution on the patterned electrode. This provides a simple way to investigate the correlation between the orientation of micro/nanowire array and the field-effect mobility, as shown in Fig. 2(d)–(f). Here, *θ* is the angle between the long axis of the micro/nanowire array and the edges of the contacts that define the physical width of the conducting channel. The decreased mobility is observed when *θ* decreases from 80° to 50°. The maximum mobility is 0.79 cm^2^V^−1^s^−1^ with the long axis of the micro/nanowire array nearly paralleling to the conducting channel (*θ* = 80°). This result can be attributed to the anisotropy of the crystal. Generally, the crystals grow preferentially in the strongest molecule stacking direction^27^. Therefore, for 1D micromolecular organic crystals, such as micro/nanowires or micro/nanobelts, their long-axis direction commonly possesses the strongest π-π stacking^28–31^. When the micro/nanowire array is grown on the source/drain electrode at a certain angle *θ*, carriers transport along the direction of the shortest distance between source and drain electrode. When *θ* = 90°, i.e., the growth of micro/nanowire array parallels to conducting channel, carriers transport along the long-axis direction of micro/nanowire where carriers possess the strongest intermolecular coupling, and hence device possesses the highest mobility in this direction. With the decreasing of the *θ* angle, the carrier transport gradually deviates from the direction of the strongest intermolecular coupling, resulting in the decreased mobility^28^, as shown in Fig. 2(d)–(f).

**Figure 3:**
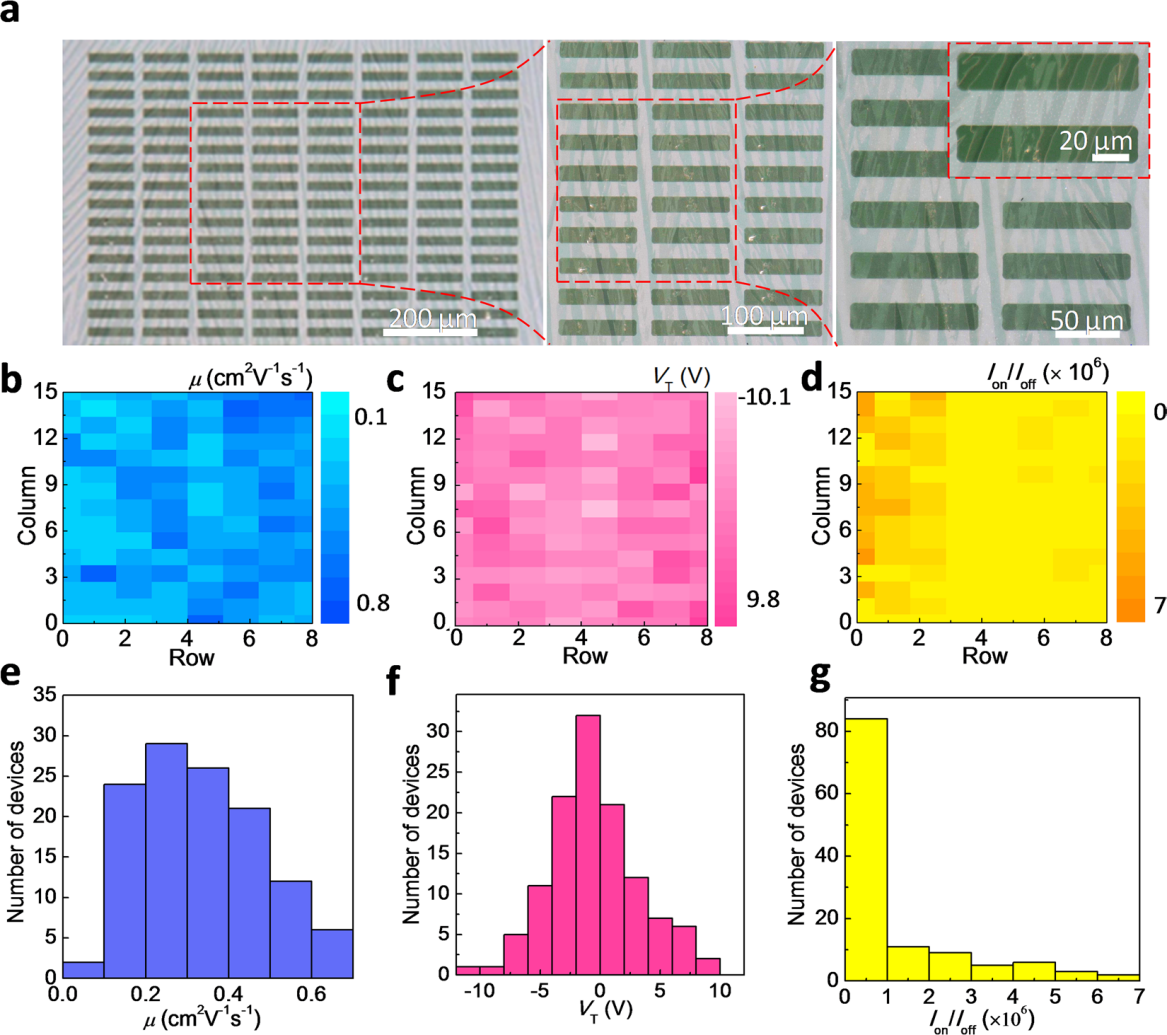
Uniformity test of 15 × 8 transistor array. (**a**) Transmission optical microscopy images of a typical TIPS-pentacene single-crystal FET array and its magnified views. Field-effect mobility (*μ*), threshold voltage (*V*_T_), and current on/off ratio (*I*_on_/*I*_off_) color maps (**b**–**d**) and distribution (**e**–**g**) of the 120 transistors.

As organic electronic devices mature into practical applications, the success rate of device fabrication and performance distribution become the crucial factors. Here, we have fabricated an TIPS-pentacene single-crystal FET array of 120 transistors over an area of 0.61 mm^2^. Its photo images are shown in Fig. 3(a). Compared to the other reported flexible transistor array which is achieved by print or shadow-mask technology^32–35^, we successfully integrated modern photolithography technology into our OFET fabrication, and then the integration level of our devices is effectively improved. Figure 3(b)–(g) show color maps and the corresponding distributions of the calculated field-effect characteristic parameters in the TIPS-pentacene single-crystal FET array. As shown in Fig. 3(b)–(d), device uniformity is nicely expressed by the spatial distribution of the transistor performance. The statistical results of Fig. 3(e)–(g) show the mobility, threshold voltage (*V*_*T*_), and current on/off ratio (*I*_on_/*I*_off_) distribution, respectively. The high yield, 100% working transistor array shows the mobility distribution where 98% of transistors have mobilities higher than 0.1 cm^2^V^−1^s^−1^. Mobility average value is 0.43 cm^2^V^−1^s^−1^ with a standard deviation of 0.15 cm^2^V^−1^s^−1^. The highest mobility reaches 0.79 cm^2^V^−1^s^−1^ in saturation regime. This value is superior to all reported bottom-contact TIPS-pentacene transistors (Table S1, Supporting Information). It is well known that the bottom-contact OFETs are favorable for integration of organic devices and circuits compared with the top-contact transistors. The small-scale source/drain electrodes and wiring can be formed in bottom-contact devices by photolithography, whereas there is a serious limit to scaling down the channel length and width with a top-contact configuration using fine metal shadow masks. 82.5% of transistors show the threshold voltages in the range of −5 to 5 V. The lowest threshold voltage is at −0.01 V, which ensures the low operating voltage. It is very important for lowered power dissipation, improved portability and security of devices. The current on/off ratio of devices is focused on 10^5^, and 30% of transistors are on the order of 10^6^. Our bottom-contact devices with excellent field-effect performance can be attributed to the coplanar configuration that eliminates the electrode step^36,37^. As shown in the cross-section image of Fig. 2(a), the embedded Au source/drain electrode in PVA facilitates the continuous growth of organic micro/nanowires in solution process. For the commonly reported solution-based bottom-contact organic micro/nanowire FETs, the source/drain electrodes protrude out of the dielectric which inevitably forms the electrode steps and hence affects the growth of micro/nanowires, resulting in the performance deterioration and even performance destruction^16,17^. In contrast, our device with the novel embedded electrodes effectively eliminates the electrode step. Therefore, the TIPS-pentacene single-crystal FET array show good uniformity, high field-effect performance and 100% success rate of device fabrication. In addition, our versatile anti-solvent conformal electrode also can integrate with other soluble organic semiconductor materials as shown in Figure S3, which show the universality of our way for the fabrication of conformal single-crystal transistors. Altogether these results demonstrate that our photolithography-compatible anti-solvent conformal electrode provides an effective way to fabricate high-performance bottom-contact transistor array, and shows the strong potential for next-generation organic electronic device.

**Figure 4:**
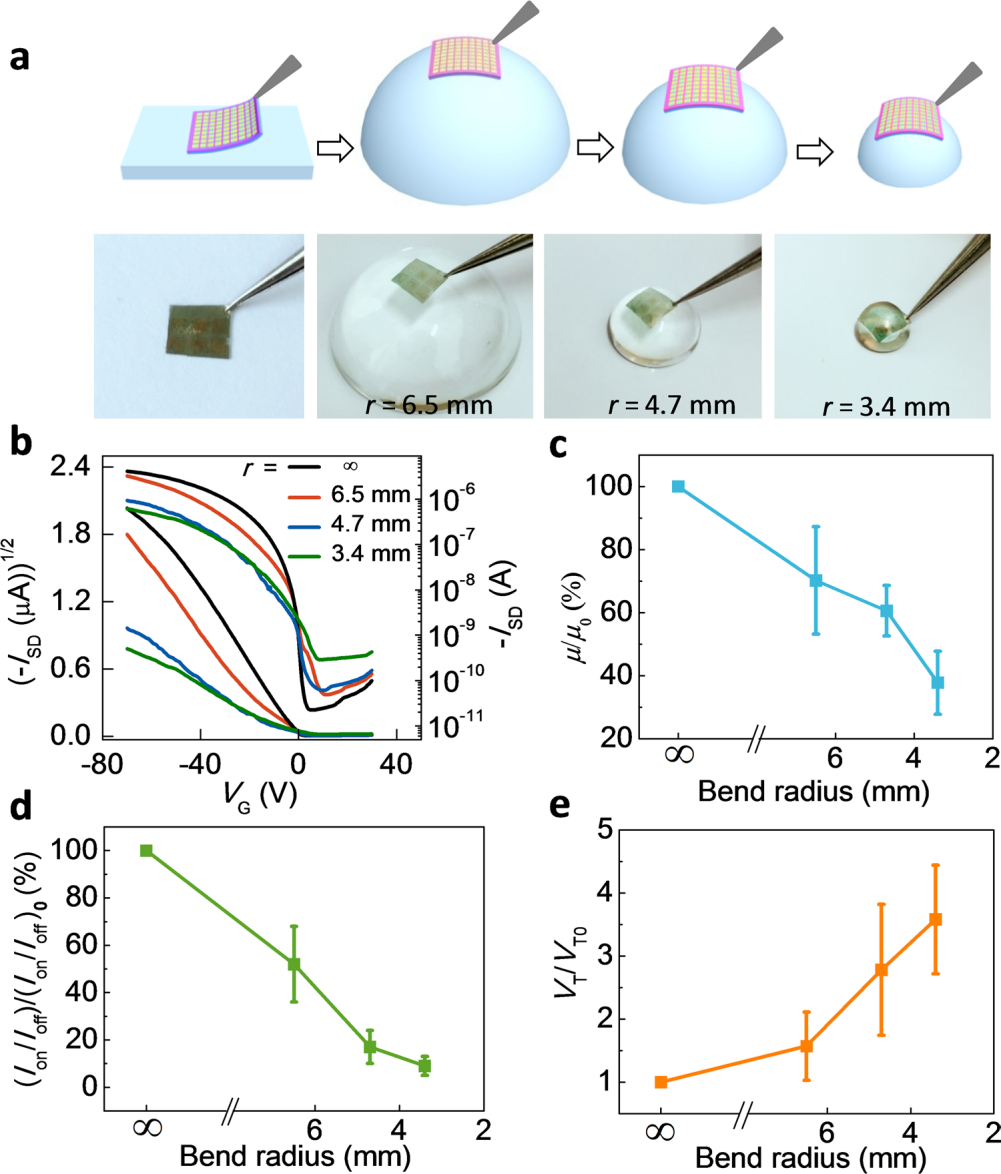
Electrical characteristics of conformal organic single-crystal FET array on glass hemispheres at different bending radius. (**a**) Schematic diagrams and the corresponding digital photos. (**b**) Typical transfer curves at different bending radius. (**c**–**e**) Dependence of the field-effect characteristic parameters, mobility (**c**), current on/off ratio (**d**), and threshold voltage (**e**), on the bending radius. *µ*_0_, (*I*_on_/*I*_off_)_0_, *V*_T0_: on flat supporting, *µ*, (*I*_on_/*I*_off_), *V*_T_: on different glass hemispheres.

To show the conformal capability of our solution-processed organic micro/nanowire transistor array onto the curved objects, we adhered the device array onto the glass hemispheres with different sizes. Figure 4(a) shows the schematic diagrams and the corresponding digital photos of the adherence process. We used a tweezer to gently peel off a device array from the plane substrate, and sequentially attach it onto glass hemispheres at different bending radius. Figure 4(b) shows transfer characteristics of a typical device adhered onto a flat substrate and three glass hemispheres with different bending radius. In the case of the minimum bending radius (*r* = 3.4 mm), the device still remains the typical p-type field-effect performance. With the decrease of the bending radius from ∞ to 3.4 mm, the slope of −*I*_SD_^1/2^ versus *V*_G_ curve shows the obviously decrease, suggesting the decreased mobility. The intersection of the fitting line and *V*_G_ axis, i.e. threshold voltage, moves to the negative direction. In order to reliably show the effect of mechanical strain on the field-effect performance, six devices were first put onto the flat Si substrate, and successively were conformed onto the glass hemispheres with different bending radius. Their electrical properties were respectively measured, and the statistical data was shown in Fig. 4(c)–(e). All devices can well operate on different-sized glass hemispheres. With the bending radius of hemisphere down to 3.4 mm, i.e. increasing strain from 0% to 0.18%^38^, the average mobility and *I*_on_/*I*_off_ decrease to 37.8% and 9%, and the average threshold voltage increases by ~3.6 times. The decreased field-effect performance can be attributed to bending induces interfacial strain on the crystal. When the device array is attached onto glass hemispheres, it is mainly subject to tensile strain, which inevitably results in the increase of the intermolecular distance of the organic semiconductor as shown in the XRD image (Figure S4), and hence reduces the HOMO level overlap by the transfer of integration leading to the decrease of the field-effect performance^39^. In contrast, when the thickness of organic single crystal is reduced to 30 nm as shown in Figure S5, the mechanical flexibility of device array will be effectively improved and the field-effect performance is almost unchanged under plane and curved surfaces.

**Figure 5:**
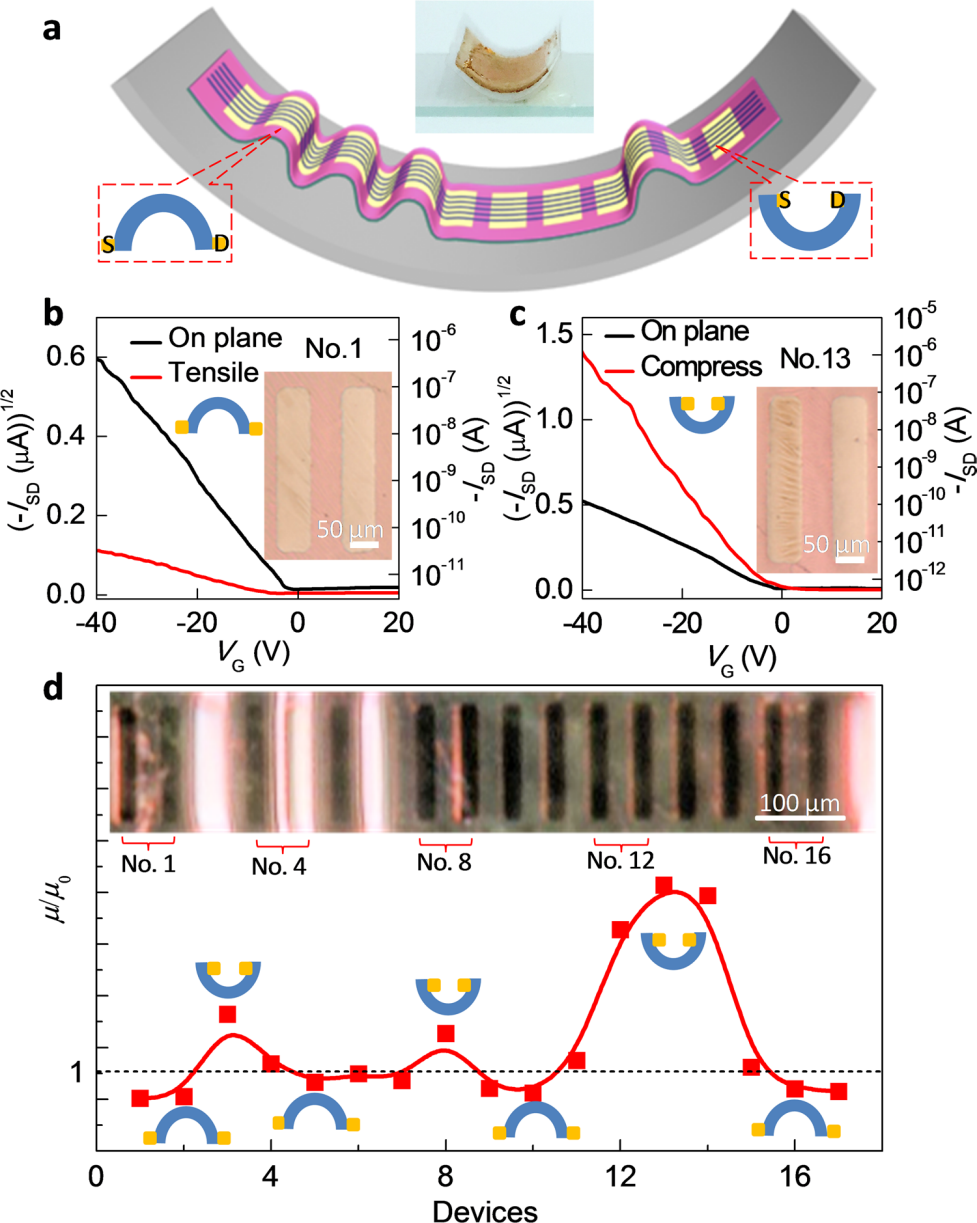
Electrical characteristics of conformal organic single-crystal FET array under different bending strain. (**a**) Schematic diagram (not to scale) and the real digital photo of a device array. The device array was unevenly attached onto a curved inner wall to deliberately form tensile and compressible states. (**b**,**c**) Typical transfer curves and the corresponding optical microscopy images under tensile state and compressible state. (**d**) Mobility changes of the device array after adherence onto the curved inner wall. *µ*_0_: mobility on flat supporting. *µ*: mobility on the curved inner wall. The inset is the corresponding 3D optical microscopy image.

To further show the strain effects on electrical properties of devices, we deliberately made a TIPS-pentacene device array unevenly attach onto a curved inner wall, that enable the entire device array to simultaneously form tensile and compressible states with different radius of curvature. Figure 5(a) shows the schematic diagram of the device (not to the scale) and the real digital photo. 16 devices in the different strain states were measured. Figure 5(b) and (c) show transfer characteristics and the corresponding photo images of two typical devices in the tensile state and compressible state, respectively. Compared with the planar electrical properties, the mobility of No. 1 device decreases to 47.5% in the tensile state, while the mobility of No.13 device dramatically increases to 650% in the compressible state. According to the mobility changes of 16 devices (Fig. 5(d)), the mobility presents decrease in the tensile state, and increase in the compressible state. The mobility change of our devices in different bending states originates from bending induced change of intermolecular distance in crystals. As mentioned above, the tensile strain inevitably results in the increase of the intermolecular distance of the organic semiconductor. On the contrary, the compressible strain will reduce the intermolecular distance, increasing the mobility^40,41^. These basic study of electro-mechanical properties of the fabricated organic single-crystal conformal FET array demonstrates that our devices can be used as an ideal tool to essentially understand how mechanical deformation affects the electrical performance, which is critical for the development of the next generation of flexible and conformal electronic devices^40,41^.

## Conclusion

In summary, based on the pre-designed anti-solvent conformal electrode, we successfully integrated solution-processed TIPS-pentacene single-crystal micro/nanowire array into conformal FETs. This novel fabrication technique of organic FET array possesses many outstanding advantages such as integration of photolithography technology into organic electronics, low process temperature, and high field-effect performance. Devices show outstanding electrical properties with device yield as high as 100%, field-effect mobility up to 0.79 cm^2^V^−1^s^−1^, low threshold voltage, and good device uniformity. Our investigation opens up the capability of solution-processed organic crystal array for applications in large-scale, low-cost, conformal electronics.

## Methods

### Anti-solvent Embedded Laminated Electrodes Fabrication

(i) Si substrates modified by a self-assembled layer of octadecyltrichlorosilane (OTS) (Acros, 95%), were employed for the carrier chip during the experiments. The OTS treatment was proceeded by dipping the Si wafers into OTS solution (OTS: heptane = 1:1000 by volume) for 3 h. (ii) Au patterns were prepared on OTS modified Si wafers by a lift-off photolithography process. Prior to photoresist remove, the sample was exposed to 3-mercaptopropyltrimethoxysilane (MPT) (Sigma-Aldrich, 95%) under vacuum for 20 min. (iii) The embedded gate electrode was fabricated by spin coating PDMS (Dow Corning, Sylgard 184) on MPT modified Au electrodes. To fabricate embedded source/drain electrodes, PVA (Sigma-Aldrich, average M_W_ ~ 205000 g/mol) was spin coated on MPT modified Au electrodes. PVA was dissolved in deionized water at a concentration of 60 g/L. The measured dielectric capacitance was ~ 1 × 10^−8^ Fcm^−2^. (iv) The gate electrode embedded in PDMS supporting layer was peeled from OTS modified Si wafer. The source/drain electrodes embedded in PVA dielectric and the gate electrodes embedded in PDMS were respectively placed in a plasma oxidation chamber and oxidized for 100 s. The soft and elastic embedded laminated electrode was obtained by peeling off the whole structure (source/drain/gate electrodes, PVA dielectric, and PDMS supporting layer) from the OTS/Si wafer. The electrode was flipped over and its peeled surface was used to grow solution-processed organic semiconductor for transistor fabrication.

### Growth of TIPS-pentacene micro/nanowire arrays

A droplet of TIPS-pentacene/chloroform solution was cast onto the prefabricated anti-solvent conformal electrode. The thickness of the whole device array is ~10 μm. As the solvent evaporation, the large-scale TIPS-pentacene micro/nanowire FET array could be successfully obtained. The highest mobility is obtained by optimization of solvents and concentration of TIPS-pentacene (Sigma-Aldrich, ≧99%). The optimized results show the good orientation and uniform TIPS-pentacene single-crystal array can be form on the anti-solvent conformal electrode with 0.3 g/L TIPS-pentacene/ chloroform solution. The growth of TIPS-pentacene micro/nanowire arrays were carried out at room temperature under atmospheric condition.

### Characterization

The 3D optical microscopy images of TIPS-pentacene micro/nanowire FET array were obtained by 3D digital microscope (Hirox, KH-8700). Optical microscopy images were obtained with an Olympus BX51, and the electrical characteristics of FET devices were recorded with a Keithley 4200 SCS and a Cascade M150 probe station in a clean and shielded box at room temperature in air.

## Electronic supplementary material

Supporting Information video

Supporting Information
