# Supplementary material for: Conformal transistor arrays based on solution-processed organic crystals
Source: Sci Rep. 2017 Nov 13;7:15367. doi: 10.1038/s41598-017-15518-y (PMC5684343; doi:10.1038/s41598-017-15518-y)
Supplement: Supplementary file 2 — Supporting Information [file 41598_2017_15518_MOESM2_ESM.docx]

Supporting Information

**Conformal transistor arrays based on solution-processed organic crystals**

Xiaoli Zhao, Bing Zhang, Qingxin Tang*, Xueyan Ding, Shuya Wang, Yuying Zhou, Yanhong Tong, and Yichun Liu*

Key Laboratory of UV Light Emitting Materials and Technology under Ministry of Education, Northeast Normal University, Changchun 130024, P. R. China

*E-mail: tangqx@nenu.edu.cn; ycliu@nenu.edu.cn

Tel./fax: +86-431-85099873.


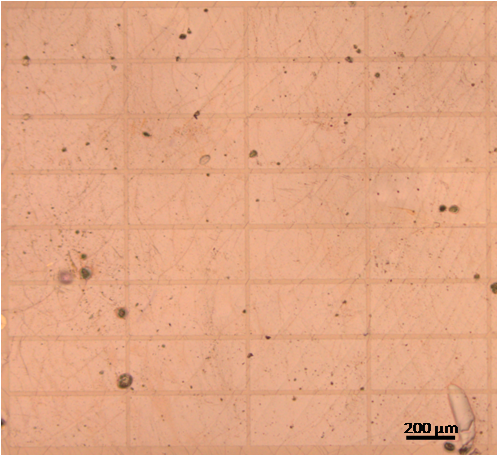


**Figure S1. Optical microscopy image of the growth of TIPS-pentacene crystals on PDMS surface.** It was found that the swelling of PDMS occurred when organic solution was drop-casted onto PDMS embedded laminated electrode, resulting in the formation of only few crystal arrays.

**Figure S2.** Mechanical property of PVA film. (a) Peeling off a PVA layer from OTS/Si substrate. Adhering the PVA layer onto (b) glass hemisphere and (c) human finger.


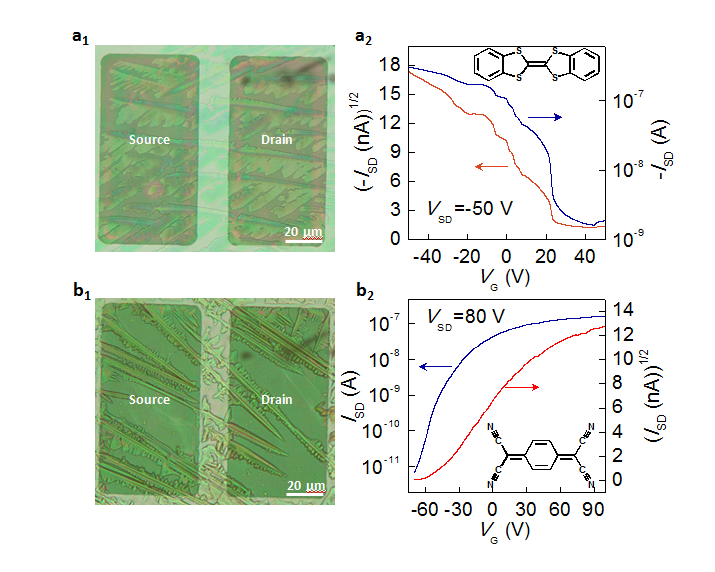


**Figure S3.** Transmission optical microscopy images and the corresponding typical transfer curves of DB-TTF and TCNQ conformal single-crystal FETs. (a_1_,a_2_) DB-TTF. (b_1_,b_2_) TCNQ.

**Figure R4.** XRD characterization of the TIPS-pentacene conformal single-crystal FETs under (a) plane and (b) glass hemisphere surface with the bending radius of 3.4 mm.

According to the formula: 2*d*sin*θ* = *kλ*, where *d* is intermolecular distance, *λ* is the wavelength of X ray and *k* is the diffraction series, intermolecular distance *d* increases when the *θ* angle decreases. Therefore, the device array showed the decrease of the field-effect performance when it was attached onto glass hemispheres.

**Figure S5.** The mechanical flexibility of different thickness of TIPS-pentacene single-crystal array. AFM images and the corresponding transfer curves on plane and glass hemispheres at 4.7 mm bending radius with the thickness of (a_1_,a_2_) 100 nm and (b_1_,b_2_) 30 nm.

As shown in Fig. S5a_1_, when chloroform is used as the solvent of TIPS-pentacene, the obtained thickness of TIPS-pentacene single crystal is ～100 nm. The corresponding transfer curves on plane and glass hemisphere at 4.7 mm bending radius are shown in Fig. S5a_2_, and the mobility shows obvious decrease. In contrast, when n-hexane is used as the solvent, the thickness of TIPS-pentacene can be reduced to 30 nm (Fig. S5b_1_), and its mobility is almost unchanged adhered onto the 4.7 mm glass hemisphere (Fig. S5b_2_).

**Table S1.** Detailed performance list for the reported bottom-contact TIPS-pentacene field-effect transistors.

| **Semiconductor** | **Mechanical properties** | **Supporting layer** | **Mobility (cm^2^V^-1^s^-1^)** | **Threshold voltage (V)** | **Current on/off ratio** | **Ref.** |
| --- | --- | --- | --- | --- | --- | --- |
| TIPS-pentacene | Rigid | Si | 0.002 | - | 10^4^ | 1 |
| TIPS-pentacene | Rigid | Si | 0.007 | -15 | - | 2 |
| TIPS-pentacene | Rigid | Si | 0.016 | - | - | 3 |
| TIPS-pentacene | Rigid | Si | 0.076 | -0.7 | 10^4^ | 4 |
| TIPS-pentacene | Rigid | Si | 0.35 | 2.9 | >10^4^ | 5 |
| TIPS-pentacene | Rigid | Glass | 0.37 | 1.08 | 3.86×10^5^ | 6 |
| TIPS-pentacene | Flexible | Mylar | 0.4 | - | 10^4^ | 7 |
| TIPS-pentacene | Rigid | Si | 0.53 | 0.13 | 10^7^ | 8 |
| TIPS-pentacene | Flexible | Plastic | 0.54 | 2.2 | 10^4^ | 9 |
| TIPS-pentacene/PS^a)^ | Flexible | PEN^b)^ | 0.6 | 3 | 10^6^ | 10 |
| TIPS-pentacene | Conformal | PDMS^c)^ | 0.79 | -0.01 | 6.82×10^6^ | Our work |

^a)^polystyrene

^b)^polyethylene naphthalate

^c)^Polydimethylsiloxane

1. B. Wang, T. Zhu, L. Huang, T. L. D. Tam, Z. Cui, J. Ding & L. Chi, *Org. Electron.* **24**, 170 (2015).
2. J. G. Park, R. Vasic, J. S. Brooks & J. E. Anthony, *J. Appl. Phys.* **100**, 044511(2006)
3. N. Onojima, N. Nishio & T. Kato, *Jpn. J. Appl. Phys.* **52**, 05DB06 (2013)
4. S. Basu, F. Adriyanto & Y. H. Wang, *Nanotechnology* **25**, 085201(2014).
5. M. Shao, S. Das, K. Xiao, J. Chen, J. K. Keum, I. N. Ivanov, G. Gu, W. Durant, D. Li & D. B. Geohegan, *J. Mater. Chem. C* **1**, 4384 (2013).
6. J. S. Kim & C. K. Song, *Thin Solid Films* **589**, 620 (2015).
7. H. T. Yi, M. M. Payne, J. E. Anthony & V. Podzorov, *Nat. Commun.* **3**, 1259 (2012).
8. K. Sakamoto, K. Bulgarevich & K. Miki, *Jpn. J. Appl. Phys.* **53**, 02BE01 (2014).
9. B. Kang, N. Park, H. Min, J. Lee, H. Jeong, S. Baek, K. Cho & H. S. Lee, *Adv. Electron. Mater.* **1**, 1500301 (2015)
10. L. Feng, W. Tang, J. Zhao, R. Yang, W. Hu, Q. Li, R. Wang & X. Guo, *Sci. Rep.* 2016, **6**, 20671.

**Supporting Information Movies**

**Movie S1.** A movie of dipping a PVA substrate into chloroform solution. the PVA film do not produce any dissolved or swollen phenomenon.
